# Different Clinical Questions Need Different Estimands

**DOI:** 10.1007/s43441-026-01012-z

**Published:** 2026-07-07

**Authors:** Oliver Keene, Helle Lynggaard, Christine Fletcher, David Wright, Paul Terrill

**Affiliations:** 1 KeeneONStatistics, Maidenhead, UK; 2 Biostatistics, Novo Nordisk A/S, Bagsværd, Denmark; 3 Biostatistics, GlaxoSmithKline Plc, Stevenage, UK; 4 Statistical Innovation, Respiratory and Immunology Biometrics and Statistical Innovation, Biopharmaceuticals R&D, AstraZeneca, Cambridge, UK; 5 PT Stat Consulting Ltd, Church Stretton, UK

**Keywords:** Estimands, Treatment policy, Hypothetical strategy, Causal inference, Intercurrent events

## Abstract

The ICH E9(R1) estimands framework requires precise specification of the treatment effects (estimands) a trial is designed to estimate. A recent article by Troxel et al. [1] has advanced a narrow view that only estimands using the treatment-policy strategy are scientifically defensible. In particular, the article recommends that journals adopt a new policy with regard to reporting results from clinical trials, advocating that only results based on the treatment policy strategy should appear in the main body of the paper with estimates based on other strategies relegated to supplementary materials. Treatment-policy estimands target the effect of assignment to treatment and are defined to include outcomes after non-terminal post-randomization events, such as treatment discontinuation or initiation of alternative or rescue medications. Use of this estimand requires that outcome collection continues following these intercurrent events. In the presence of missing data, estimation of effects using the treatment policy strategy typically relies on strong, unverifiable assumptions [2]. While using treatment policy strategies to address all intercurrent events is appropriate for certain scientific objectives, these estimands do not address all clinically relevant questions. Results based on estimands using alternative strategies for primary and key secondary objectives should therefore also be presented in the main body of the published paper when they address important clinical questions that are relevant to patient care or decision-making.

## Different Questions, Different Effects

The PIONEER-1 trial, [3] which compared semaglutide with placebo for glycemic control in Type 2 diabetes, illustrates this point. Rescue medication is routinely given to patients with persistent hyperglycemia, and additional glucose-lowering therapies could also be prescribed after discontinuation of randomized treatment. Use of such therapies was substantially more frequent among placebo recipients than semaglutide recipients: 19.7% in the placebo group compared with 9.1%, 4.6% and 4.0% in the semaglutide 3-mg, 7-mg and 14-mg groups, respectively [3]. These additional therapies comprised multiple drug classes, including biguanides, sulfonylureas, insulin, DPP-4 inhibitors, and thiazolidinediones.

Several distinct questions arise. Is the treatment effect of interest the effect of initiating semaglutide, potentially followed by different glucose-lowering medications, compared to placebo potentially followed by different glucose-lowering? Or is it the effect of semaglutide itself versus placebo without the use of these additional medications? Similarly, is the estimand of interest the effect of assignment to semaglutide ignoring whether the course of treatment was discontinued, or the effect that would be observed if patients adhered to treatment as prescribed?

Each of these important clinical questions corresponds to different causal estimands. Addressing these important clinical questions may require readily available estimates of treatment effect for the different estimands in publications reporting trial results.

## Beyond the ITT / Per-protocol Dichotomy

Historically, analyses have often been framed as a dichotomy between ITT and per-protocol analyses. These are sometimes expressed as estimating either the effect of assignment to intervention or the effect of adhering to the intervention as specified in the protocol (per-protocol effect). For example, the Cochrane Risk of Bias tool [4] describes these effects as: “(1) the effect of assignment to the interventions at baseline (regardless of whether the interventions are received during follow-up, sometimes known as the ‘intention-to-treat effect’); (2) the effect of adhering to intervention as specified in the trial protocol (sometimes known as the ‘per-protocol effect’)”. However, there are important clinical questions which are not addressed by either of these analyses [5].

Adherence to an intervention is an ambiguous concept as it may refer to compliance with the randomized treatment, adherence to a broader treatment strategy, and/or adherence to the protocol procedures. Moreover, reference to per-protocol effects can easily be confused with estimation using a per-protocol analysis set. The per-protocol analysis set typically restricts analysis only to participants who complete the assigned treatment as assigned and who have no major protocol deviations and therefore typically removes some participants with observed data from the analysis altogether, which is known to be a biased approach to estimation.

The ICH E9(R1) estimands framework resolves much of this ambiguity by requiring explicit identification of intercurrent events and specification of strategies for handling them, thereby allowing the clinical questions of interest to be precisely defined. Hernán and Robins [6] argued that randomized trials are not limited to estimating ITT effects and that restricting inference to only the treatment effect of randomisation assignment risks answering a less relevant clinical question.

As well as the treatment policy strategy, the ICH E9(R1) estimands framework describes several alternative strategies. Hypothetical, principal-stratum, and while-on-treatment strategies can all be used to estimate well-defined causal effects of treatment adherence, provided that the required assumptions are clearly stated and justified [7].

The composite strategy can provide an alternative to treatment policy by incorporating events such as initiation of rescue or additional medication directly into the endpoint definition. This approach explicitly recognises these events as clinically meaningful indicators of treatment failure. By contrast, treatment-policy includes outcomes after initiation of rescue or additional medication without accounting for the lack of efficacy that prompted their use [8].

The intercurrent event of death requires separate consideration because outcomes after death are not observable, making a treatment-policy strategy impossible for that intercurrent event. A composite strategy may be appropriate for death, although the choice of strategy should be guided by the scientific question of interest.

## Hypothetical Strategy and Reality

Troxel et al. [1] state that an effect estimate based on a hypothetical strategy is “often of limited value because the scenario didn’t occur in reality.” This criticism conflates *counterfactual* with *irrelevant*.

The fact that a clearly articulated hypothetical scenario did not fully occur during the trial does not diminish its scientific or clinical value. Rather, such estimands explicitly address questions that trials are often designed to inform but cannot directly observe. By contrast, treatment-policy estimands may reflect a composite of downstream interventions and patient behaviors that obscure the effect attributable to the randomized treatment itself. Estimands targeting the causal effect of adhering to treatment therefore often provide information that is more directly relevant to patients and prescribers and may also inform regulatory decision making when understanding the efficacy attributable to a specific medicine rather than assignment alone, while facilitating more meaningful comparisons across trials. For example, the European regulatory (CHMP) guideline for diabetes describes using the hypothetical strategy that rescue medication was not initiated for participants who take it [9]. This addresses the clinical question of what the treatment effect would have been in the absence of rescue medication.

## Assumptions are Unavoidable

Troxel et al. [1] express concern that estimation use of hypothetical estimands requires assumptions that “*generally cannot be verified or refuted with available data*”. In the presence of missing data, estimation of effects using the treatment policy strategy is also difficult and also typically relies on assumptions that cannot be verified with available data.

For a treatment policy estimand, every attempt should be made to collect data after intercurrent events to minimise issues relating to missing data. However, nearly all clinical trials will continue to encounter missing data and in some therapeutic areas—such as psychiatry—retention is particularly challenging, leading to extensive post-discontinuation missing data that must be imputed. Troxell et al. state that estimation of treatment-policy estimands must therefore proceed under “*appropriate context-specific assumptions”.* It is unclear how to determine the “appropriate context-specific assumptions” for a participant’s outcome given that once they have left the trial the participant could receive interventions which are often heterogeneous, poorly characterized, and influenced by clinical judgment. This is a considerably more complicated problem than determining the best projection had they continued to take the assigned treatment and there may be a shortage of observed data within the trial to allow estimation that conditions fully on all interventions taken.

Bell and colleagues [10] note that while treatment policy estimands are easy to specify they are hard to estimate reliably when the primary outcomes of interest are not observed for all trial participants. Clinical researchers face a dilemma between making strong assumptions that risk bias or weaker assumptions that lead to imprecision and loss of power.

The relevant comparison, therefore, is not between assumption-free and assumption-laden estimands, but between assumptions that are explicit and aligned with the clinical question, and assumptions that are implicit and poorly understood.

## Clinical Relevance and Pluralism

Troxel et al. [1] caution against misinterpretation of analyses that target estimands other than treatment policy and recommend de-emphasizing them in primary publications. We agree that clear definition and clinical explanation are essential. However, restricting the main body of the publication to simply results for estimands based on the treatment policy strategy may not align with trial objectives. For example, sponsors, in discussion with regulatory agencies and following clinical guidance documents, may prespecify estimands that use strategies other than treatment policy as primary estimands to address key regulatory questions particularly when the goal is to understand the efficacy attributable to the intervention itself rather than the effect of assignment alone. If the recommendation of Troxel et al. [2] that journals publish only results from estimands using treatment policy strategies in the main body of the paper, then a pre-specified primary estimand and its associated analysis, agreed in advance with the European regulatory body, could be relegated to the supplementary materials.

Different stakeholders reasonably prioritize different questions, and the estimands framework was designed to accommodate this diversity. A pluralistic approach, reporting multiple well-justified estimands with transparent assumptions, can provide additional insights.

## Conclusion

Randomized clinical trials can answer multiple causal questions. Provided those questions are explicitly and precisely defined, estimands and estimators can be aligned with each question, bringing transparency to the assumptions required for estimation of the treatment effect. Randomization identifies the causal effect of assignment, but it does not restrict investigators to that effect alone. Rather than treating estimands other than treatment policy as secondary or misleading, they should be recognized as legitimate and often essential complements. These alternative estimands can provide clinically meaningful information for clinicians and patients deciding whether, and how, to use a medicine and for regulators to decide whether to license a medicine. Consequently, there should be no requirement for journals to limit the main text of publications to only results based on the treatment policy strategy.

## Data Availability

No datasets were generated or analysed during the current study.
